# Is It a Pathogenic *ATP7A* Variation and Is It Menkes Disease?

**DOI:** 10.1155/2015/358605

**Published:** 2015-06-07

**Authors:** Zeynep Tümer

**Affiliations:** Applied Human Molecular Genetics, Kennedy Center, Department of Clinical Genetics, Rigshospitalet, Copenhagen University Hospital, Gammel Landevej 7, Glostrup, 2600 Copenhagen, Denmark

This paper is a comment on “Menkes Disease Presenting with Epilepsia Partialis Continua.” While updating the LOVD (Leiden Open Variation Database)* ATP7A* mutation database (https://grenada.lumc.nl/LOVD2/MD/home.php) I came across a case described by Rizk and colleagues and published in your journal [1].

Rizk and colleagues report a 17-month-old girl who was referred to their hospital with continuous right hand movements which developed suddenly at age of 14 months, regression of milestones, and repetitive episodes of hypothermia. She had hypotonia and constipation. Brain magnetic resonance imaging showed among others cortical and cerebral atrophy. EEG was abnormal and she was diagnosed with epilepsia partialis continua. Magnetic resonance angiography showed relatively tortuous but patent intracranial vessels, with appearance of “hair pin” sign. She had normal hair also with light microscopy. Blood copper and ceruloplasmin levels were normal. The patient was born full term and had normal birth measures. Genetic testing revealed presence of a heterozygous variation, c.1138G>A [p.(Val380Met)], and the authors concluded the diagnosis of Menkes disease (MD) in this patient.

Even though the clinical features of this female patient overlap with some of the features observed in Menkes disease patients, she lacks some of the major pathogenic criteria (such as reduced serum copper and ceruloplasmin levels, abnormal hair, hypopigmentation, and cutis laxa) (review in [2]) questioning the clinical diagnosis of Menkes disease, even though female patients may be less severely affected. Furthermore, the* ATP7A* variation identified is not convincing enough to establish a genetic diagnosis.

Firstly, this variation is described at least once in a healthy individual (rs149523862, dbSNP database of short genetic variations, http://www.ncbi.nlm.nih.gov/SNP/), which questions its pathogenicity especially when taking into consideration that MD is a Mendelian disorder. This variation is a missense substitution in exon 4 of* ATP7A* proximal to but not within the 4th MBD (metal binding domain) of the ATP7A protein. Until now 70 different missense mutations in* ATP7A* have been described in 94 unrelated patients and none of these mutations were within the first 7 exons of* ATP7A* encoding the six MBDs [3]. It is generally believed that variations within these regions are more acceptable and do not necessarily lead to MD [3]. Furthermore, two commonly used and publicly available pathogenicity prediction methods, PhD-SNP (http://snps.biofold.org/phd-snp/phd-snp.html) and nsSNP analyser (http://snpanalyzer.uthsc.edu/), categorize this variation as neutral. Secondly, MD is an X-linked recessive disorder and manifesting heterozygotes are rare [4]. It is therefore extremely important to investigate the inheritance of this mutation and to perform X-inactivation studies before evaluating the clinical effect of this variation.

In conclusion, the clinical diagnosis of this patient and the pathogenicity of the identified* ATP7A* variation should be reevaluated.
